# Study on the Resistance of Concrete to High-Concentration Sulfate Attack: A Case Study in Jinyan Bridge

**DOI:** 10.3390/ma17143388

**Published:** 2024-07-09

**Authors:** Yingda Zhang, Zhaopeng Tang, Xinyue Liu, Xianliang Zhou, Wenting He, Xiaojun Zhou

**Affiliations:** 1 School of Architecture and Civil Engineering, Xihua University, Chengdu 610039, China; yingda.zhang@xhu.edu.cn (Y.Z.);; 2 Centre for Infrastructure Engineering and Safety, School of Civil and Environmental Engineering, The University of New South Wales, Sydney, NSW 2052, Australia

**Keywords:** sulfate attack, sulfate erosion inhibitors, concrete performance enhancement, high-sulfate-acid environment, sulfate resistance

## Abstract

Concrete structures face significant challenges in sulfate-rich environments, where sulfate attack can affect their durability and structural integrity. This study explores innovative approaches to enhancing concrete performance by integrating hydrophobic and densification technologies. It emphasizes the critical role of anti-sulfate erosion inhibitors in mitigating sulfate-induced damage, reducing water absorption, and inhibiting corrosive reactions. This research addresses prevalent issues in Chinese engineering projects where high sulfate concentrations are common, necessitating robust solutions for sulfate resistance. Through rigorous testing, including wet–dry cycling tests with 5% and 10% Na_2_SO_4_ solutions following the GB/T 50082-2009 standard, concrete formulations achieved exceptional long-term sulfate resistance, meeting or exceeding KS200-grade requirements. These findings provide valuable insights into optimizing concrete durability in sulfate-rich environments, offering practical strategies to enhance infrastructure resilience and reduce maintenance costs.

## 1. Introduction

Concrete durability refers to its ability to withstand various environmental factors while maintaining its performance and structural integrity throughout its service life [1,2]. Durability is a crucial indicator of concrete quality, directly influencing the lifespan and safety of buildings and infrastructures [3]. Key factors affecting concrete durability include climatic conditions, chemical attack, freeze–thaw cycles, carbonation, and physical abrasion, etc. [4,5]. High-durability concrete must possess low permeability, crack resistance, and strong chemical resistance to safeguard against the ingress and damage caused by external harmful substances [6]. For instance, in saline or sulfate-rich environments, concrete must effectively resist the attack of chloride ions or sulfates to prevent steel corrosion and concrete expansion or cracking [7]. Therefore, enhancing concrete durability is essential for extending the lifespan of buildings and infrastructure, reducing maintenance costs, and improving overall structural safety and stability.

The investigation of concrete resistance to sulfate attack is of paramount importance. Sulfate erosion is a prevalent form of deterioration in reinforced concrete structures exposed to sulfate environments, particularly in areas with sulfate-rich groundwater, seawater, and industrial wastewater [8]. Extensive research indicates that when reinforced concrete is subjected to sulfate solutions, it undergoes complex physical, chemical, and mechanical degradation [9]. This process involves the generation of expansion stress from chemical reactions, internal pressure from physical crystallization, and the softening of cement paste, resulting in a loss of its binding capacity. Consequently, microcracks develop within the concrete, leading to gradual degradation. Once sulfate erosion causes concrete cracking, harmful chemicals from the environment can penetrate the interior of the concrete more rapidly, accelerating carbonation and steel corrosion, thereby rapidly weakening the concrete mechanical properties [10]. Therefore, a comprehensive understanding of concrete behavior in sulfate environments, along with the development of concrete mixes and construction methods with enhanced sulfate resistance, is crucial for extending the service life of reinforced concrete structures, reducing maintenance costs, and ensuring the safety of engineering projects.

To address the issue of low-concentration sulfate attack on concrete, several strategies can be implemented. For instance, adjusting the water–cement ratio to a lower value can significantly reduce the porosity of concrete, thereby decreasing the permeability of sulfate solutions [11]. Additionally, utilizing high-quality aggregates and cement enhances the compactness and erosion resistance of the concrete. The inclusion of mineral admixtures, such as fly ash, silica fume, and slag powder, improves the concrete microstructure and increases its density, ultimately enhancing its sulfate resistance [12,13]. These admixtures, through pozzolanic reactions, generate secondary hydration products that fill the pores in the concrete, further enhancing its durability [14].

Furthermore, selecting specifically designed sulfate-resistant cements, such as sulfate-resistant cement or sulfoaluminate cement, which are optimized in their formulation and production processes, can significantly enhance the concrete performance against sulfate attack [15,16]. Applying protective coatings, such as waterproof paints, sealants, or silane impregnations, to the concrete surface effectively blocks the penetration of sulfate solutions, thereby minimizing erosion [17]. Moreover, these coatings also improve the concrete abrasion resistance and freeze–thaw durability. By employing these methods, the durability of concrete in low-concentration sulfate environments can be effectively improved, extending its service life and ensuring the safety and stability of the structure.

Moreover, the use of anti-sulfate erosion inhibitors plays a crucial role in mitigating the effects of sulfate erosion [18]. These inhibitors work by chemically interacting with sulfate ions, reducing their reactivity and thus preventing the formation of expansive and damaging compounds within the concrete matrix [19]. The addition of such inhibitors into concrete mixes can significantly extend the service life of reinforced concrete structures, reduce maintenance costs, and ensure the structural integrity and safety of engineering projects.

Meanwhile, the complex engineering geological conditions in Western China, characterized by harsh environments such as high salinity, significant temperature variations, and low humidity, pose significant challenges to the durability of infrastructure in resisting external environments [20]. During the investigation of various engineering projects in Zigong, Chengdu, Leshan, Nanchong, and Liangshan Prefecture in Sichuan Province of China, it was discovered that there exists a mirabilite layer underground, with SO_4_^2−^ concentrations in the sampled soil reaching up to 136,627.2 mg/L (and in some areas, even exceeding 633,104 mg/L). These values are ten times higher than the maximum limits specified in existing regulations [21]. Consequently, engineering practice in these high-concentration sulfate environments has already begun, underscoring the urgent need to explore the failure characteristics and degradation processes of concrete under ultra-high sulfate concentrations [22]. Research focusing on these specific environmental conditions is crucial to developing effective treatment techniques and ensuring the long-term durability and stability of infrastructure in such challenging settings.

In summary, the main objective of this study is to investigate the preparation of concrete resistant to high concentrations of sulfate attack, aiming to address the technical challenges associated with the fabrication and application of concrete in environments with extremely high sulfate concentrations. This paper also studies the effect of anti-corrosion inhibitors on conventional concrete and sulfate-resistant cement (SRC)-based concrete. This research is innovative in its integration of sulfate attack inhibitors to enhance concrete durability in such harsh conditions. Furthermore, this study provides valuable insights for the evaluation and design of concrete durability against high sulfate concentrations, offering practical solutions and advancing the current understanding in this critical area of materials science and engineering.

## 2. Experimental Program

### 2.1. Materials

**Figure 1:**
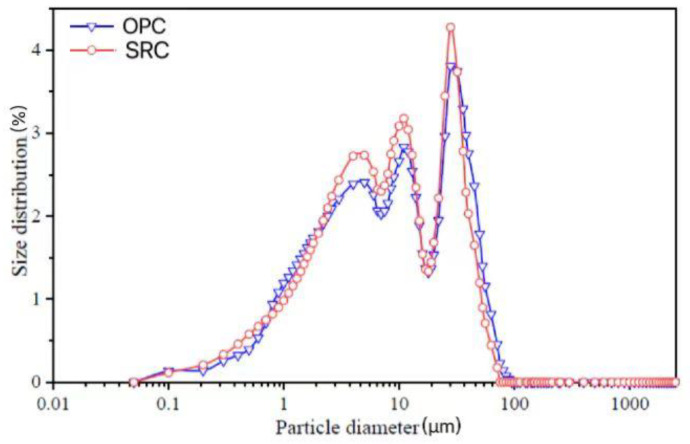
Particle size distribution of OPC and SRC.

The experimental materials used in this research project included ordinary Portland cement (OPC) with a strength grade of 42.5 MPa from Dujiangyan Lafarge Cement Co., Ltd. (Chengdu, China). The sulfate-resistant cement (SRC) used was a medium-grade sulfate-resistant cement manufactured by Shandong Huayin Special Cement Co., Ltd. (Zibo, China). The particle size distribution of OPC and SRC is presented in Figure 1. Class II fly ash (FA) was sourced from Emei Hongyuan Resource Recycling Development Co., Ltd. (Emeishan, China), in accordance with GB/T 1596-2017 [23], and silica fume (SF) from Chengdu Keliang Building Materials Co., Ltd. (Chengdu, China), complying with GB/T 18736-2017 [24]. The reason to use fly ash and silica fume in this study is that they can react with Ca(OH)_2_ in cement hydration products, consume part of Ca(OH)_2_, and reduce the relative content of Ca(OH)_2_ in cement paste. The alkalinity of aqueous solution in the pores of cement paste is reduced, which greatly decreases the speed and amount of crystallization of ettringite and gypsum, thereby enhancing the corrosion resistance of concrete. The chemical compositions of the above binders tested by XRF are presented in Table 1. The fine aggregate used was machine-made sand from Chongzhou, with an apparent density of 2630 kg/m^3^, a bulk density of 1640 kg/m^3^, a fineness modulus of 2.9, and a stone powder content of 5.0%. For coarse aggregate, Chongzhou crushed stone with a continuous gradation of 5–25 mm was utilized, with an apparent density of 2660 kg/m^3^, a bulk density of 1610 kg/m^3^, and a mud content of 0.5%. 

The admixture employed was polycarboxylic acid water-reducing agent (WRA) from Zigong Xingxing Chemical Building Materials Co., Ltd. (Zigong, China), with a water-reducing rate of 25.4%. The anti-erosion inhibitor SBT-TIA was from Jiangsu Sobote New Materials Co., Ltd. (Nanjing, China), which was added by replacing an equivalent volume of water. This inhibitor meets the performance requirements of the Chinese standard JC/T 2553-2019 [25]. It contains amphiphilic groups, where the hydrophobic functional groups physically alter the surface tension of the concrete’s internal capillaries, significantly reducing the water absorption rate and enhancing the hydrophobicity of the cement paste. This action inhibits the leaching of calcium ions from the hardened cement paste in erosive environments. Meanwhile, the hydrophilic groups chelate with Ca^2+^ in the pore solution, forming nanoparticles that block the concrete gel pores, hindering the transport of media. Consequently, this effectively enhances the sulfuric acid corrosion resistance of reinforced concrete. The anhydrous sodium sulfate was sourced from Chengdu Kelong Chemical Co., Ltd. (Chengdu, China), with a Na_2_SO_4_ content exceeding 99%.

### 2.2. Mix Design

This study employed seven distinct mix proportions to investigate the sulfate resistance of concrete, as shown in Table 2. According to the trail mix results, the compressive strength of concrete met the construction requirements when the water–binder ratio was 0.34. Mixes 1 and 2 examine the effects of single-component admixtures, specifically fly ash (designated as OPC-FA) and a combination of fly ash and silica fume (designated as OPC-SF). Mixes 3, 4, and 5 focus on the influence of varying inhibitor dosages on sulfate resistance, with the mixes labeled OPC-SF-T15, OPC-SF-T20, and OPC-SF-T25, respectively. In mixes 6 and 7, this study assessed sulfate resistance using sulfate-resistant cement (SRC), both with and without inhibitor admixtures. These mixes are designated as SRC-SF and SRC-SF-T20 based on the cement type, respectively. Additionally, comparative analyses were conducted between mixes 2 and 4 to evaluate the impact of inhibitor use on sulfate resistance in both sulfate-resistant cement and ordinary Portland cement. Moreover, the water-reducing admixture was kept consistent and controlled at 1.65% initially. However, a good workability of some concrete mixes could not be achieved. As such, the dosage of the water-reducing admixture was slightly adjusted according to the actual situation. The initial slump of all concrete mixes was controlled within 235 mm–245 mm, and the concrete had good encapsulation and fluidity.

### 2.3. Compressive Strength Test

According to GB 50010-2010 [26], the compressive strength of seven different groups was tested at 3, 7, and 28 days. The compressive strength of various concrete samples was evaluated, and the strength value for each group represents the average of three test specimens.

### 2.4. Cyclic Immersion Corrosion Test

The primary objective of this study was to validate the sulfate resistance of concrete prepared using the proposed technical scheme through corrosion tests involving wet–dry cycles in sulfate solutions [27]. Furthermore, this study aimed to investigate the impacts of various mineral admixtures, inhibitor dosages, and types of cement on the sulfate resistance of concrete. Given the high concentration of sulfate ions in the soil environment within the pile foundation of this engineering project, which surpasses the standard sulfate ion concentration specified in relevant codes, this research incorporated both a 5% Na_2_SO_4_ solution, as outlined in the Chinese Standard GB/T 50082-2009 [28], and an additional 10% Na_2_SO_4_ solution for accelerated corrosion tests to comprehensively assess the sulfate resistance of the concrete.

**Figure 2:**
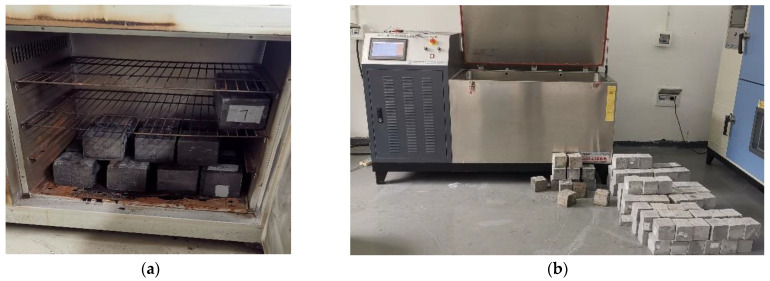

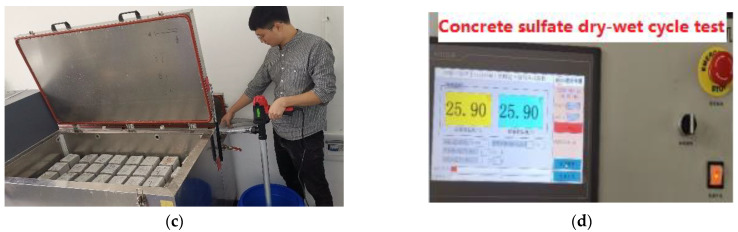
Test procedures. (**a**) Oven-dry process. (**b**) Preparation of the test chamber. (**c**) Liquid drainage from the storage tank. (**d**) Wet–dry cycling immersion test.

According to the sulfate resistance test method outlined in Chinese Standard GB/T 50082-2009 [28], specimens were molded and cured for 26 days out of the total 28-day aging period, as depicted in Figure 2. Two days prior to the 28th day, specimens designated for wet–dry cycling were removed from the standard curing chamber. These specimens were then dried by removing surface moisture and subsequently placed in an oven at (80 ± 5 °C) for 48 h. After drying, the specimens were cooled to room temperature in a dry environment. Subsequently, the specimens underwent immersion in a sulfate wet–dry cycling chamber, where each wet–dry cycle lasted for 24 ± 2 h. Upon completing the specified number of wet–dry cycles, the specimens were removed and their compressive strength was tested. Concurrently, the compressive strength of concrete specimens cured under standard conditions for the same duration was also tested. Finally, the sulfate resistance coefficient of the concrete was calculated using Equation (1) to determine the concrete sulfate resistance grade.

(1) $$K_{f}=\frac{f_{cn}}{f_{c0}}\times 100$$

where $K_{f}$ represents the compressive strength corrosion resistance coefficient expressed as a percentage (100%); $f_{cn}$ denotes the compressive strength test value (in MPa, accurate to 0.1 MPa) of a set of concrete specimens subjected to sulfate corrosion after N wet–dry cycles; while $f_{c0}$ refers to the compressive strength test value (in MPa, accurate to 0.1 MPa) of a set of reference concrete specimens that underwent standard curing for the same age as the sulfate-corroded specimens.

## 3. Test Results and Discussion

### 3.1. Compressive Strength Test Results

**Figure 3:**
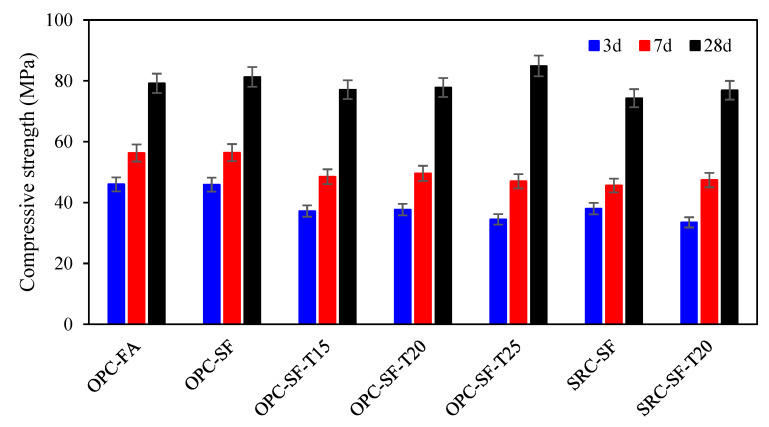
Compressive strength of concrete.

As depicted in Figure 3, under identical conditions, the early-age strength of concrete employing sulfur-resistant cement is lower than that of ordinary Portland cement concrete. Specifically, the 3-day strength of SRC-SF and SRC-SF-T20 is lower by 7.9 and 4.2 MPa, the 7-day strength by 10.8 and 2.2 MPa, and the 28-day strength by 7.0 to 1.1 MPa than that of OPC-SF and OPC-SF-T20, respectively. This is due to the utilization of sulfate-resistant cement, which has a lower content of C_3_A compared to OPC, and the controlled content of C_3_S, both of which are designed to minimize the formation of ettringite, thus resulting in a reduced early strength [29].

Through the adjustment of the inhibitor properties, both the fluidity and cohesion of the concrete have been significantly improved. The slump exceeds 235 mm, and the spread exceeds 600 mm. Additionally, the slump retention time remains stable at around 10 s, with minimal loss of workability within 3 h, thus meeting the requirements for underwater concrete placement. 

### 3.2. Cyclic Immersion Corrosion Test in 5% Na_2_SO_4_ Solution

**Figure 4:**
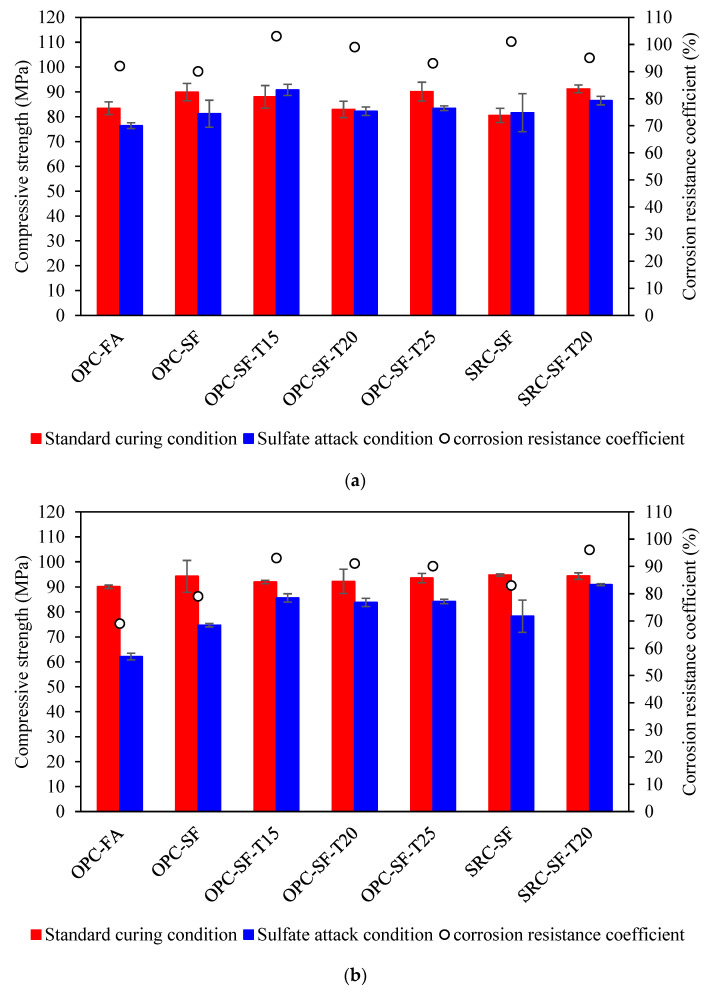

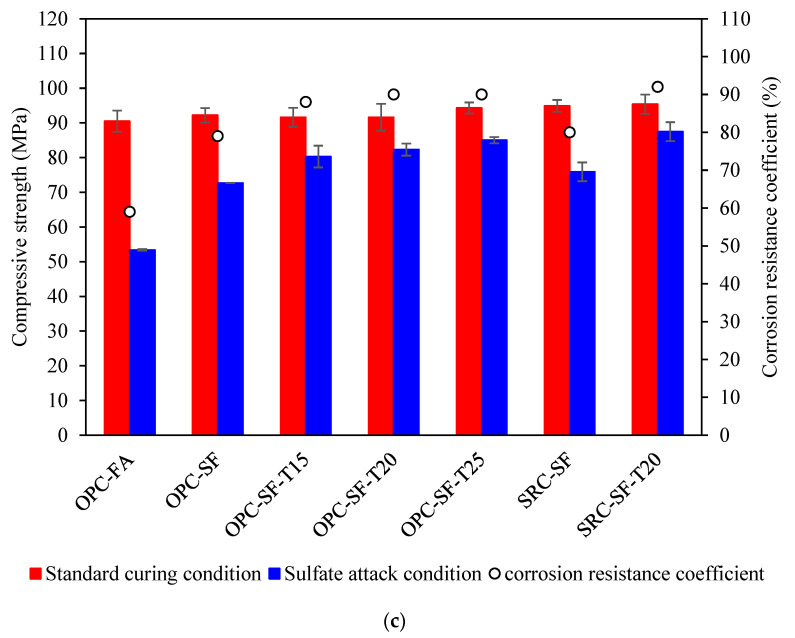
Test results using 5% Na_2_SO_4_ solution. (**a**) 60 cycles. (**b**) 150 cycles. (**c**) 200 cycles.

Figure 4a presents the test results after 60 cycles of wetting and drying experiments. It is evident that, when utilizing a 5% Na_2_SO_4_ solution, all concrete specimens exhibited a corrosion resistance coefficient of 75% or higher after 60 cycles. This finding aligns with the requirements for sulfate resistance grade KS60, as specified in the GB/T50082-2009 Standard [28]. 

Comparative analysis of the test results for OPC-SF, OPC-SF-T15, OPC-SF-T20, and OPC-SF-T25 reveals that the KS60 values are 90%, 103%, 99%, and 93%, respectively. Notably, the incorporation of anti-erosion inhibitors leads to a significant enhancement in the KS60 corrosion resistance coefficient compared to specimens without inhibitors. This is attributed to the bifunctional nature of the sulfate resistance inhibitor. Its hydrophobic functional groups, through physical interactions, modify the surface tension of the capillary pores within the concrete, significantly reducing its water absorption rate. This enhancement in the cement paste hydrophobicity suppresses the leaching of calcium ions from the hardened cement paste in erosive environments, thereby improving the concrete compactness and hydrophobicity [30]. However, an initial decrease in the corrosion resistance coefficient is observed with increasing inhibitor dosage. Furthermore, Figure 4a highlights that without inhibitors, the corrosion resistance coefficient of SRC-SF is higher than that of OPC-SF. However, after adding 20 kg of inhibitors per cubic meter, OPC-SF-T20 exhibited a higher corrosion resistance coefficient compared to SRC-SF-T20. This could be attributed to the relatively low total content of C_3_A and C_3_S in sulfate-resistant cement, which leads to a slower reaction rate of the cement hydration products, thereby affecting the development of early strength. The use of inhibitors may not fully counteract the negative effects caused by the reduced content of C3A and C3S [31]. The corrosion resistance coefficients of OPC-SF-T20 and SRC-SF are roughly equivalent, indicating a comparable level of early-stage sulfate erosion resistance. 

After 150 cycles of wetting and drying, the test results are presented in Figure 4b. Except for OPC-FA, all other specimens maintain a corrosion resistance coefficient above 75%, satisfying the KS150 requirements. Following 150 cycles, the corrosion resistance coefficient of compressive strength for different binder systems shows a decline compared to the 60-cycle test, with OPC-SF decreasing to 79%, while OPC-FA is lower than 75%, failing to meet the requirements of the GB/T50082-2009 Standard [28]. When the amount of fly ash admixture is excessive, it can lead to an excessive reduction in the amount of calcium hydroxide in the concrete, thereby decreasing the alkalinity of the concrete. Since the rate of sulfate attack is typically correlated with the alkalinity of the concrete, a decrease in alkalinity may lower the concrete resistance to sulfate erosion [32]. 

Notably, OPC-SF-T15, OPC-SF-T20, and OPC-SF-T25 exhibit minimal changes in their corrosion resistance coefficient of compressive strength after 150 cycles compared to 60 cycles, whereas OPC-SF experiences a slight reduction. This indicates that the addition of anti-erosion inhibitors helps maintain the sulfate resistance of concrete. Comparing OPC-SF-T20 with SRC-SF-T20, and OPC-SF with SRC-SF, it is evident that the specimens incorporating anti-erosion inhibitors exhibited significantly higher corrosion resistance coefficients of compressive strength after 150 wetting–drying cycles than those without inhibitors. Furthermore, the combination of sulfur-resistant cement and anti-erosion inhibitors synergistically enhances the sulfate resistance of concrete.

The test results after 200 cycles of wetting and drying are shown in Figure 4c. Following the 200 cycles, except for OPC-FA, all other specimens maintain a corrosion resistance coefficient above 75%, meeting the KS200 requirements. Notably, OPC-SF remains largely unchanged at 79%. This further underscores that the combination of fly ash and silica fume provides superior sulfate resistance to concrete compared to using fly ash alone [33].

As illustrated in Figure 4c, the corrosion resistance coefficient of compressive strength for OPC-SF-T15, OPC-SF-T20, and OPC-SF-T25 decreases after 200 cycles compared to the 150-cycle mark. However, the decrease is less significant with higher dosages of inhibitors. Evidently, increasing the dosage of anti-erosion inhibitors enhances the long-term sulfate resistance of concrete.

Comparing OPC-SF-T20 with SRC-SF-T20 and OPC-SF with SRC-SF reveals that the specimens with anti-erosion inhibitors exhibited significantly higher corrosion resistance coefficients of compressive strength after 200 wetting–drying cycles than those without inhibitors.

### 3.3. Cyclic Immersion Corrosion Test in 10% Na_2_SO_4_ Solution

**Figure 5:**
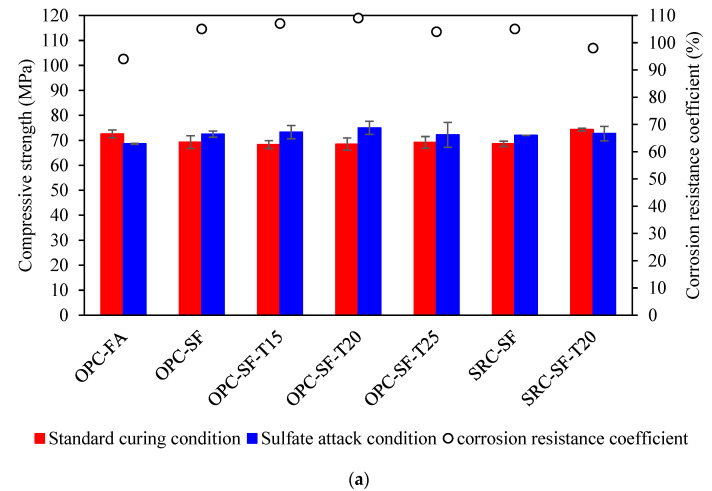

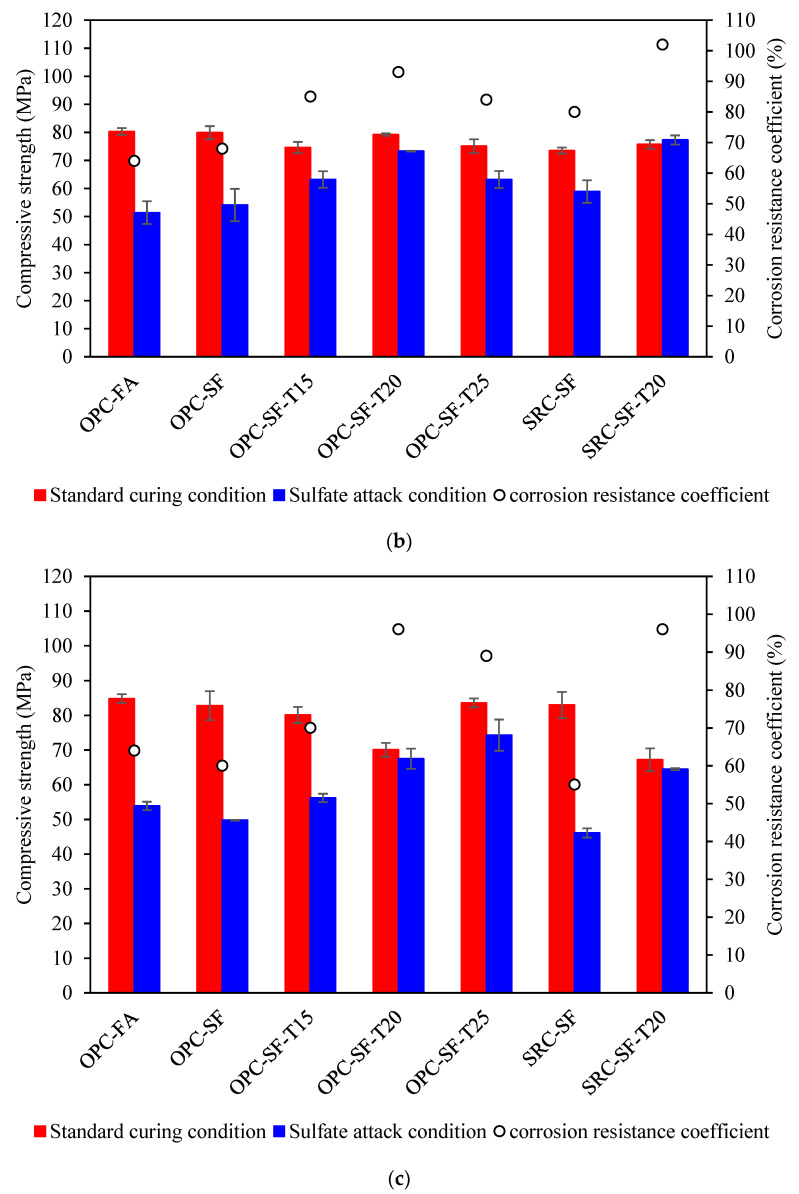
Test results of 200 wet–dry cycles using 10% Na_2_SO_4_ solution. (**a**) 60 cycles. (**b**) 150 cycles. (**c**) 200 cycles.

The results after conducting 60 cycles of wet–dry tests are presented in Figure 5a. It is evident that when utilizing a 10% Na_2_SO_4_ solution, all concrete specimens exhibit a compressive strength durability factor of 75% or higher after undergoing 60 wet–dry cycles. This aligns with the requirements specified in GB/T50082-2009 [28], for resisting sulfate attack under 60 cycles of wet–dry exposure in a 10% Na_2_SO_4_ solution.

As observed in Figure 5a, following 60 days of standard curing, the compressive strength of concrete specimens under different cementitious material systems increased. This is because in a short period of time, high concentrations of sodium sulfate react with calcium hydroxide in cement to generate highly dispersed calcium sulfate. This substance has a large specific surface area and can quickly react with calcium aluminate in cement to generate ettringite, which expands in volume, thereby increasing the strength of the concrete. Among those subjected to 10% Na_2_SO_4_ solution wet–dry cycles for the same duration, OPC-SF concrete with silica fume addition exhibits an improved compressive strength, whereas OPC-FA exhibits a decrease in compressive strength after 60 cycles. This suggests that OPC-SF concrete, when exposed to 10% Na_2_SO_4_ solution and 60 wet–dry cycles, demonstrates a significant enhancement in its early-age compressive strength durability factor compared to OPC-FA. 

When incorporating inhibitors, the compressive strength of concrete after 60 wet–dry cycles in a 10% Na_2_SO_4_ solution increased by approximately 3 to 6 MPa compared to that of the same-age specimens under standard curing conditions. Compared to the compressive strength durability factor without inhibitor addition, the durability factor of concrete subjected to 60 wet–dry cycles in 10% Na_2_SO_4_ solution exhibits an initial increase and then a subsequent decrease with increasing inhibitor dosage. However, the overall durability factor remains higher than that of OPC-SF without inhibitors. Notably, when the inhibitor dosage is 15, 20, and 25 kg/m^3^, the compressive strength durability factor reaches 107%, 109%, and 105%, respectively. Additionally, it is observed that without inhibitors, SRC-SF shows a comparable durability factor to OPC-SF. However, adding 20 kg/m^3^ of inhibitors, OPC-SF-T20 displays a higher durability factor compared to SRC-SF-T20. This suggests that the early-age durability factor of OPC concrete is enhanced by the addition of inhibitors, while the durability factor of SRC concrete decreases.

The test results after 150 wet–dry cycles are depicted in Figure 5b. It is evident that all concrete specimens except OPC-FA and OPC-SF maintain a corrosion resistance coefficient of 75% or higher after undergoing 150 wet–dry cycles. This adheres to the requirements of GB/T50082-2009 [28], for resisting sulfate attack under 150 cycles of wet–dry exposure.

After 150 days of standard curing, a slight increase in the compressive strength of concrete under different cementitious material systems is observed. However, under the same duration of 10% Na_2_SO_4_ solution wet–dry cycles, the compressive strength of concrete under different cementitious material systems starts to decrease significantly, with a more pronounced decline in the compressive strength durability factor. Specifically, OPC-FA and OPC-SF concretes experience a reduction in their compressive strength durability factor to 64% and 68%, respectively, after 150 wet–dry cycles, falling below the standard limit of 75% and failing to meet the requirements for resisting sulfate attack under 150 wet–dry cycles.

Concrete with inhibitor addition also experiences a decrease in compressive strength after 150 wet–dry cycles, but the overall decline is relatively small. Compared to the compressive strength durability factor without inhibitors, as the inhibitor dosage increased, the compressive strength durability factor of concrete under 150 wet–dry cycles in 10% Na_2_SO_4_ solution initially rose and then declined. Specifically, a dosage of 20 kg/m³ of inhibitors resulted in a maximum compressive strength durability factor of 93%. This indicates that the inhibitor exhibits stable performance in enhancing the long-term resistance of concrete to high-concentration sulfate wet–dry cycles. Currently, the use of high-dosage mineral admixtures has shifted from enhancing structural compactness through sulfate attack-induced expansive products to causing stress damage within the internal structure, manifesting as a significant decline in concrete compressive strength after sulfate wet–dry cycles. This ultimately leads to a marked reduction in the compressive strength durability factor, making it difficult to meet the requirements for resisting sulfate attack under 150 wet–dry cycles [34].

Without inhibitor addition, SRC-SF exhibited a lower durability factor compared to ordinary OPC-SF, possibly due to the expansive products generated by sulfate attack on ordinary Portland cement, which enhance the compactness of the concrete matrix and thereby increase the durability factor. However, after adding 20 kg/m³ of inhibitors, OPC-SF-T20 and SRC-SF-T20 exhibit comparable durability factors. The incorporation of inhibitors enhanced the durability factor in both SRC and OPC systems to a certain extent, outperforming other current technical solutions.

The test results after 200 wet–dry cycles are presented in Figure 5c. It is evident that when utilizing a 10% Na_2_SO_4_ solution, the mixes consisting of sulfate-resistant cement and inhibitor-doped specimens (OPC-SF-T20, OPC-SF-T25, and SRC-SF-T20) meet the requirements of GB/T50082-2009, for resisting sulfate attack under 200 cycles of wet–dry exposure. However, the compressive strength durability factors of the other groups fell below 75%, thus failing to satisfy the standards for resisting sulfate attack under 200 wet–dry cycles. This is because in the long term, when the sodium sulfate content is too high, it is easy to generate a large amount of ettringite quickly. The volume instability caused by excessive ettringite may lead to a decrease in concrete strength. This phenomenon is particularly obvious in the long-term period, because the ettringite generated later will destroy the microstructure inside the concrete.

As depicted in Figure 5c, following 200 days of standard curing, the compressive strength of concrete under different cementitious material systems shows a slight increase. However, under the same duration of 10% Na_2_SO_4_ solution wet–dry cycles, the compressive strength of concrete under different cementitious material systems continued to decline, with a more significant drop in the compressive strength durability factor. OPC-FA and OPC-SF concretes, after undergoing 200 wet–dry cycles in 10% Na_2_SO_4_ solution, experienced a reduction in their compressive strength durability factor to 64% and 68%, respectively, both falling below the standard limit of 75%. This indicates that, under a high-sulfate-concentration environment, simply enhancing the compactness of the matrix can only satisfy early-stage resistance to high sulfate concentrations, but long-term resistance cannot be achieved through this approach [35].

Ordinary Portland cement and sulfate-resistant cement, when mixed with silica fume and fly ash, exhibited compressive strength durability factors below 75% after 200 wet–dry cycles. However, with the addition of 20 kg/m³ of inhibitors, the compressive strength durability factors increased significantly from 60% to 96% and 55% to 96%, respectively, following 200 wet–dry cycles in 10% Na_2_SO_4_ solution.

## 4. Discussion

### 4.1. Influence of SCMs

**Figure 6:**
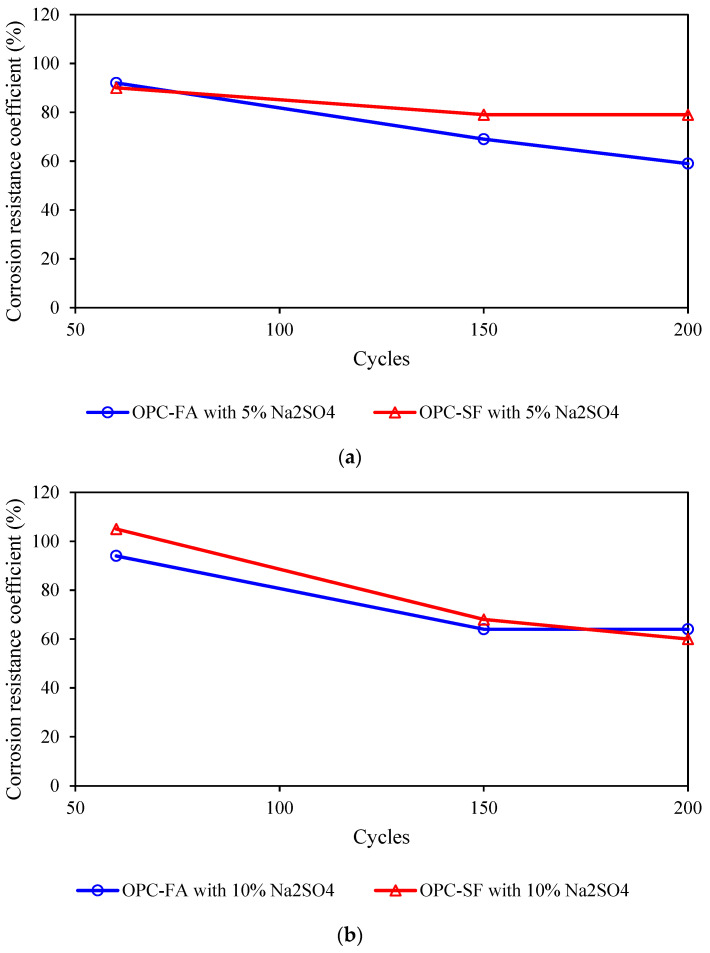
The influence of the adhesive material system on the variation in corrosion resistance coefficient. (**a**) 5% Na_2_SO_4_. (**b**) 10% Na_2_SO_4_.

Under varying dry–wet cycles in a 5% Na_2_SO_4_ solution, the impact of the adhesive material system on the sulfate resistance of concrete is illustrated in Figure 6a. Specifically, OPC-FA exhibits a notable decrease in corrosion resistance coefficient with increasing dry-wet cycles, meeting only the KS60 grade requirement. Conversely, OPC-SF, which incorporates both fly ash and silica fume, achieves a KS200 grade, demonstrating a more gradual decline in corrosion resistance coefficient and superior stability against sulfate attack. This is attributed to the fact that silica fume has a fineness and specific surface area approximately 80 to 100 times greater than those of cement, enabling it to fill the gaps between cement particles effectively, resulting in a denser concrete material. This increase in density significantly reduces the penetration of water and harmful ions, and similar results can be found in [36]. Additionally, silica fume undergoes a pozzolanic reaction with calcium hydroxide, forming calcium silicate cementitious material. This reaction diminishes the likelihood of sulfate reacting with calcium hydroxide to produce calcium sulfate and water, thereby reducing the risk of sulfate attack [37].

As for the 10% Na_2_SO_4_ solution, Figure 6b depicts the influence of the adhesive material system on the sulfate resistance of concrete after different dry–wet cycles. By incorporating highly reactive mineral admixtures, the compactness of the concrete matrix is enhanced, significantly improving its resistance to high-concentration sulfate attack in the early stages. However, as the concrete is corroded by sulfates over time, resulting in internal structural damage, the mix degrades more rapidly, and the sulfate resistance decreases significantly. In engineering projects with high sulfate concentrations, the utilization of reactive mineral admixtures such as fly ash and silica fume necessitates a more rigorous consideration of their long-term sulfate resistance performance.

### 4.2. Influence of Erosion Inhibitor

**Figure 7:**
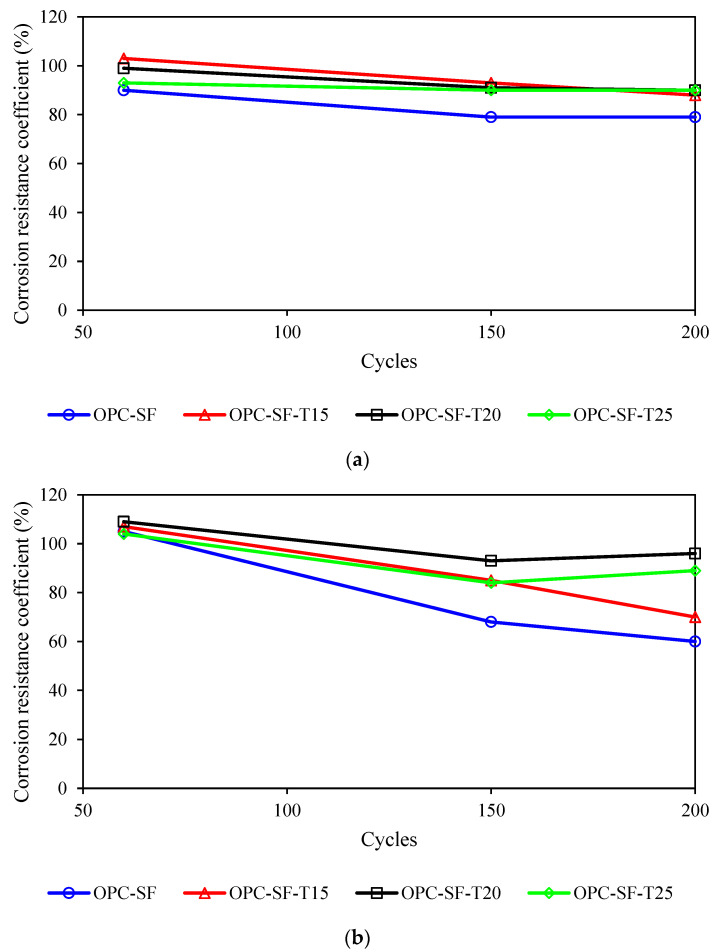
The effect of inhibitor dosage on the variation in corrosion resistance coefficient. (**a**) 5% Na_2_SO_4_. (**b**) 10% Na_2_SO_4_.

The influence of varying erosion inhibitor dosages on the sulfate resistance of concrete, subjected to different wetting–drying cycles in a 5% Na_2_SO_4_ solution, is depicted in Figure 7a. The OPC-SF, which is not reinforced with an inhibitor, exhibits a faster decline in its corrosion resistance coefficient compared to OPC-SF-T15, OPC-SF-T20, and OPC-SF-T25, all of which are treated with inhibitors. A comparative analysis of the corrosion resistance coefficients among the three inhibitor-doped specimens reveals that, despite a lower initial coefficient when the inhibitor dosage is 25 kg, the coefficient remained stable with only a slight decrease upon increased wetting–drying cycles. Consequently, a higher dosage of inhibitors correlates positively with the concrete long-term sulfate resistance, particularly under conditions of high sulfate concentration, necessitating a suitable increase in the inhibitor dosage. This is because the interaction between inhibitors and mineral admixtures results in stress-induced compaction of the concrete. The secondary hydration of mineral admixtures further optimizes the pore structure, ultimately enhancing the early strength and compactness of the concrete [38].

Figure 7b illustrates the impact of varying anti-erosion inhibitor dosages on concrete sulfate resistance after multiple wetting–drying cycles in a 10% Na_2_SO_4_ solution. It can be observed that in the 60 cycles, when 15, 20, and 25 kg of anti-erosion inhibitors are added, compared with ordinary concrete, the corrosion resistance coefficient increased by 1.9% and 3.8% and decreased by 1%, respectively, and the effect of anti-erosion inhibitors was not obvious. This may be because the added anti-erosion inhibitor takes a certain amount of time to fully penetrate the concrete, the nanoparticles or structural changes formed by the anti-erosion inhibitor inside the concrete need to gradually accumulate, and these processes may be slow in the early stages. Therefore, the early improvement in corrosion resistance is not significant. However, after the long-term period, the corrosion resistance coefficients of OPC-SF-T15, OPC-SF-T20, and OPC-SF-T25 increased by 25%, 36.8%, and 23.5%, and 16.7%, 60%, and 48.3% for 150 and 200 cycles, respectively. This is due to the gradual accumulation of nanoparticles or structural changes formed inside the concrete by anti-corrosion inhibitors over a long period of time, significantly improving the microstructure of the concrete. These improvements include refining pores, blocking capillary pores, improving compactness, etc., thereby effectively improving the corrosion resistance of concrete.

Moreover, OPC-SF, without an inhibitor, displays a faster reduction in corrosion resistance coefficient compared to OPC-SF-T15, OPC-SF-T20, and OPC-SF-T25, all containing inhibitors. Among the three inhibitor-doped specimens, the corrosion resistance coefficient is notably higher for the 20 kg dosage, maintaining stability with a minimal decline over increasing wetting–drying cycles. Therefore, under conditions of high sulfate concentration, it is advisable to exercise reasonable control over the inhibitor dosage for effective sulfate erosion mitigation. 

### 4.3. Influence of Types of Cement

**Figure 8:**
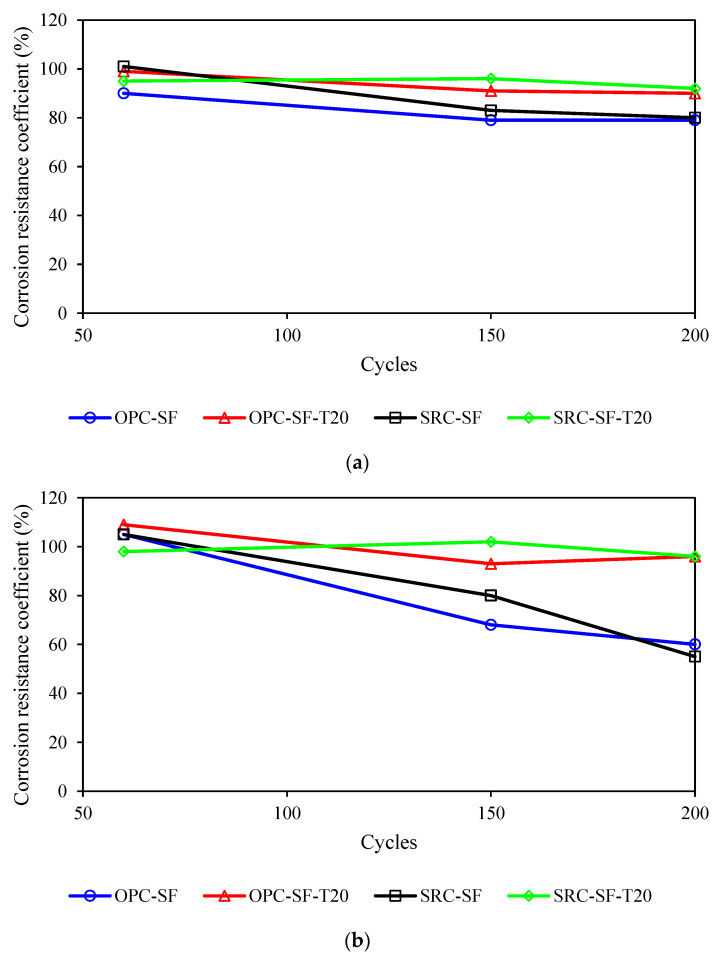
The impact of cement type on the variation pattern of corrosion resistance coefficient. (**a**) 5% Na_2_SO_4_. (**b**) 10% Na_2_SO_4_.

As depicted in Figure 8a, the impact of cement type on the sulfate resistance of concrete under varying dry–wet cycles in a 5% Na_2_SO_4_ solution is analyzed. Notably, the corrosion resistance coefficient of sulfate-resistant cement exceeds that of ordinary Portland cement. However, OPC-SF and SRC-SF, which are not blended with inhibitors, exhibit a lower corrosion resistance coefficient compared to their counterparts OPC-SF-T20 and SRC-SF-T20, which incorporate inhibitors. OPC-SF and SRC-SF possess sulfate resistance performance only up to the KS200 level. In contrast, OPC-SF-T20 and SRC-SF-T20, after enduring 250 dry–wet cycles, maintain a corrosion resistance coefficient above 80%, showcasing their exceptional sulfate resistance properties.

As shown in Figure 8b, the influence of cement type on the sulfate resistance of concrete after varying dry–wet cycles in a 10% Na_2_SO_4_ solution is examined. Notably, the corrosion resistance coefficient of sulfate-resistant cement is consistently higher than that of ordinary Portland cement. However, OPC-SF and SRC-SF, without inhibitors, display lower corrosion resistance coefficients compared to their counterparts OPC-SF-T20 and SRC-SF-T20, which incorporate inhibitors. OPC-SF and SRC-SF demonstrate sulfate resistance performance that meets the requirements for only 150 and 200 dry–wet cycles, respectively. In contrast, OPC-SF-T20 and SRC-SF-T20 maintain a corrosion resistance coefficient above 90% even after 200 dry–wet cycles, exhibiting superior resistance to high-concentration sulfate attack. 

From Figure 8, it can also be observed that both OPC and SRC can withstand 200 wetting–drying cycles in 5% Na_2_SO_4_ solution. However, under 10% Na_2_SO_4_ solution, their resistance decreases to around or below the 75% limit after 150 wetting–drying cycles, failing to meet the requirement of enduring 200 cycles. When 20 kg/m^3^ of corrosion inhibitors is added to both cement types, they exhibit good long-term sulfate resistance. Nevertheless, considering that OPC already exhibits remarkable long-term erosion resistance in high-concentration sulfate environments, the use of sulfate-resistant cement, which is not only more costly but also has limited procurement sources, becomes unnecessary when an anti-erosion inhibitor is incorporated [39].

### 4.4. Application in Jinyan Bridge Project

Within the construction site of Jinyan Bridge, argillaceous gypsum rock poses a significant corrosive threat to concrete structures and causes minor corrosion to steel reinforcement within reinforced concrete structures. The evaluation of environmental corrosiveness and permeable soil strata on concrete structures revealed SO_4_^2−^ concentrations at three sampling points, 136,396.8 mg/L, 128,332.8 mg/L, and 136,627.2 mg/L, under alternating wet and dry conditions. According to JTG/T3310-2019 [21], these conditions are classified as extremely severe for salt crystallization (V-F) and chemical corrosion (IV-F). Therefore, the urgent implementation of anti-sulfate corrosion inhibitors is necessary for structural concrete in sulfate attack-prone areas of the Jinyan Bridge project to mitigate potential sulfate-induced damage.

**Figure 9:**
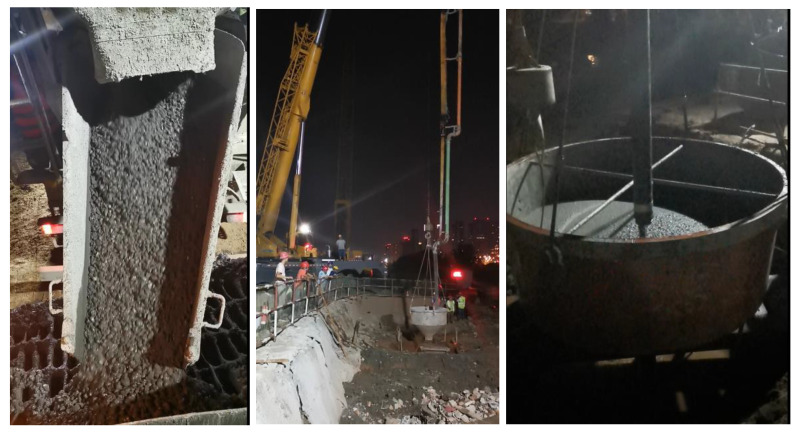
Onsite pumping and placement of concrete.

Through extensive trials and testing, the OPC-SF-T20 mix design is selected for enhancing the concrete resistance to high-concentration sulfate attack at the Jinyan Bridge. To ensure the compactness of the concrete microstructure, a C50 concrete grade is specified. The tested workability and mechanical properties are detailed in Table 3. Upon arrival, a site slump and spread test is performed on each truckload of concrete before placement to ensure compliance with the requirements prior to casting, as illustrated in Figure 9.

**Figure 10:**
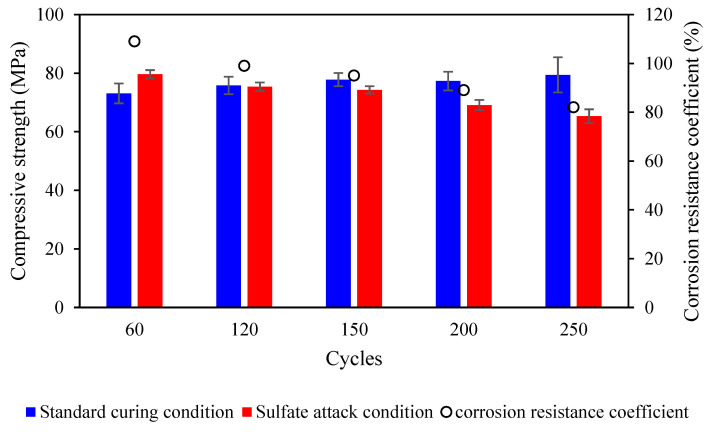
The test results of sulfate resistance performance for concrete samples taken from the site.

Concrete is obtained during onsite casting and molded in accordance with the GB/T 50082-2009 standard [28]. These samples are subjected to wet–dry cycling using a 5% Na_2_SO_4_ solution to evaluate their sulfate resistance, and the results are depicted in Figure 10. The data clearly show that the tested concrete demonstrates outstanding long-term sulfate resistance, meeting the KS250 grade requirements.

## 5. Conclusions

This study explores the integration of hydrophobic and densification technologies in concrete matrices. Hydrophobicity is achieved through the incorporation of anti-erosion inhibitors, which notably reduce water absorption rates and inhibit sulfate transport and erosive reactions. Simultaneously, densification of the concrete matrix is attained by incorporating advanced reactive mineral admixtures, thereby significantly enhancing the erosion resistance of concrete in sulfate-corrosive environments. The key conclusions are summarized as follows:

(1) In a 5% Na_2_SO_4_ solution, OPC-FA concrete did not meet the standard requirements but achieved KS200 grade when combined with silica fume. After 200 cycles, corrosion resistance did not meet the standards. The OPC-SF showed stable corrosion resistance coefficients. In a 10% Na_2_SO_4_ environment, OPC-SF met requirements for 60 cycles but dropped below 75% after 200 cycles.
(2) In a 5% Na_2_SO_4_ solution, SRC combined with fly ash and silica fume shows slightly higher corrosion resistance coefficients compared to ordinary Portland cement with the same admixtures. In a high-concentration 10% Na_2_SO_4_ environment, SRC-SF meets the requirements for 60 cycles but significantly declined after 200 cycles, failing standards. OPC-SF only meets 60-cycle requirements and fell short of 150 cycles. Thus, while OPC, fly ash, and silica fume suffice under current specifications, sulfate-resistant cement offers better performance. Yet, even with these additives, the long-term sulfate resistance of concrete in high-sulfate environments needs improvement.
(3) When combining ordinary Portland cement, fly ash, and silica fume, the addition of anti-erosion inhibitors notably enhanced corrosion resistance coefficients in both 5% and 10% Na_2_SO_4_ solutions during wet–dry cycling tests. As cycles increased, the corrosion resistance decays slower. Adjusting the inhibitor dosage shows an initial improvement followed by a decline per cycle, necessitating careful application in practice. Using sulfate-resistant cement with fly ash, silica fume, and anti-erosion inhibitors yielded higher and more stable corrosion resistance coefficients than with ordinary Portland cement.
(4) In high-concentration sulfate environments, concrete initially shows satisfactory sulfate resistance with high doses of mineral admixtures and traditional corrosion prevention methods. However, the corrosion resistance coefficient declines rapidly during later sulfate wet–dry cycles, with sulfate-resistant cement offering no significant improvement. This underscores that relying solely on increased density and sulfate-resistant cement does not effectively enhance long-term sulfate resistance. Instead, incorporating anti-erosion inhibitors improves concrete long-term corrosion resistance in both low- and high-concentration sulfate environments. While sulfate-resistant cement outperforms ordinary Portland cement initially, the latter already demonstrates significant long-term corrosion resistance in high-sulfate environments. Considering cost and availability limitations of sulfate-resistant cement, anti-erosion inhibitors offer a practical alternative.
(5) This study provides a method such as the application of anti-sulfate corrosion inhibitors in high-sulfate-concentration engineering construction areas. The performance of using anti-sulfate corrosion inhibitors is better than that of sulfate-resistance cement. In the future, more attention can be paid to the management of concrete and its anti-sulfate corrosion performance in the whole life cycle. As for the limitations of anti-sulfate corrosion inhibitors, when selecting inhibitors, it is necessary to make comprehensive considerations based on the actual situation of the specific project to ensure its effectiveness and applicability. It is also worth noting that sulfate attack has a significant impact over 365 days or even several years. This paper only studied 200 days in the laboratory and 250 days in the actual project. Long-term changes should be monitored in future studies.

## Figures and Tables

**Table 1:** Chemical compositions of OPC, SRC, silica fume, and fly ash (%).

Chemical Compositions	SRC	OPC	SF	FA
SiO_2_	20.41	20.31	96.08	46.59
CaO	61.91	65.50	0.21	4.98
Al_2_O_3_	3.55	4.80	0.84	38.52
SO_3_	2.22	2.10	0.51	0.66
Fe_2_O_3_	3.91	4.99	0.09	3.93
K_2_O	0.54	0.40	0.21	0.66
MgO	2.72	1.30	0.08	0.96
Na_2_O	0.91	0.15	0.21	0.20
TiO_2_	0.24	0.39	-	1.69

**Table 2:** Mix proportions of concrete (kg/m^3^).

No.	ID	OPC	SRC	FA	SF	Fine Aggregate	Coarse Aggregate	Corrosion Inhibitor	Water	WRA
1	OPC-FA	360	0	110	0	808	1062	0	160	1.70%
2	OPC-SF	360	0	80	30	792	1051	0	160	1.60%
3	OPC-SF-T15	360	0	80	30	792	1051	15	145	1.60%
4	OPC-SF-T20	360	0	80	30	792	1051	20	140	1.65%
5	OPC-SF-T25	360	0	80	30	792	1051	25	140	1.65%
6	SRC-SF	0	360	80	30	792	1051	0	160	1.60%
7	SRC-SF-T20	0	360	80	30	792	1051	20	140	1.65%

**Table 3:** Concrete workability and mechanical properties.

Slump/mm		Spread/mm		Vebe Time/s		Setting Time/h		Compressive Strength/MPa		
0	3 h	0	3 h	0	3 h	Initial setting	Final setting	3 d	7 d	28 d
230	215	595	570	8	10	9.4	14.0	44.2	53.1	65.6

## Data Availability

Data are contained within the article.
